# Effects of legal-market cannabis and alcohol on verbal learning and memory

**DOI:** 10.1007/s00213-025-06882-z

**Published:** 2025-09-25

**Authors:** Joshua L. Gowin, Vanessa Stallsmith, Katelyn Weldon, Gregory Dooley, Hollis C. Karoly

**Affiliations:** 1 Department of Radiology, School of Medicine, University of Colorado Anschutz Medical Campus, Aurora, CO USA; 2 Department of Psychology, Colorado State University, Fort Collins, CO USA; 3 Department of Environmental and Radiological Health Sciences, Colorado State University, Fort Collins, CO 80523-1601 USA; 4 Department of Psychiatry, School of Medicine, University of Colorado Anschutz Medical Campus, Aurora, CO USA

**Keywords:** Alcohol, Cannabis, Verbal memory, RAVLT, Simultaneous use

## Abstract

**Rationale:**

Widespread legalization of cannabis in the US in recent years has coincided with increasing use of alcohol and cannabis at the same time. Cannabis is thought to confer synergistic effects on alcohol intoxication, and the potential for increased cognitive impairment is a concern. Most prior co-administration studies have relied on low-THC cannabis, limiting generalizability to real-world consumption of higher-THC, legal-market cannabis.

**Objectives:**

We tested whether legal-market cannabis confers verbal learning and memory impairment beyond the effects of an acute dose of alcohol in a sample of heavy-drinking adults who regularly use cannabis.

**Methods:**

Participants (*N* = 60, 40% female) completed two laboratory sessions: an Alcohol Only session and a Cannabis + Alcohol session. At each session, participants completed the Rey Auditory Verbal Learning Test before and after alcohol/cannabis use. Linear mixed-effects models evaluated effects of substance use condition and sex on number of words recalled immediately and after a short and long delay.

**Results:**

During immediate recall, participants recalled one fewer word per trial in the Cannabis + Alcohol Post-Use condition compared to the other conditions (*p* <.001). This effect was stronger in females (*p* =.003). During long delay recall, participants recalled 1.5 fewer words in both Post-Use conditions compared to both Pre-Use conditions. No sex differences emerged for delayed recall trials.

**Conclusions:**

Legal-market cannabis was associated with acute verbal learning and memory impairments compared to alcohol alone, with females showing heightened vulnerability during initial encoding. Results highlight the risks of alcohol and cannabis co-use and underscore the importance of studying high-THC cannabis. Clinical Trials Registration: identifier NCT04998006.

**Supplementary Information:**

The online version contains supplementary material available at 10.1007/s00213-025-06882-z.

## Introduction

Alcohol and cannabis are two of the most commonly-used psychoactive substances worldwide (Carvalho et al. 2019; Kerr et al. 2023; Macha et al. 2022; Wang et al. 2024). In recent years, following the trend towards increasing legalization of recreational cannabis, a growing number of individuals report consuming these substances at the same time, such that their intoxicating effects overlap (Gonçalves et al. 2023). This is known as ‘simultaneous use’ and is associated with negative consequences (Fairlie et al. 2023; Lee et al. 2022) that may reflect combined pharmacological effects of both substances. Adverse effects of alcohol and cannabis co-use include acute cognitive and psychomotor impairment (Chait and Perry 1994; Fares et al. 2022). At present, the data are conflicting regarding the extent to which the use of cannabis alongside alcohol impairs cognition over and above the effects of alcohol alone. Some studies suggest that alcohol and cannabis confer additive or synergistic effects (Chait and Perry 1994; Downey et al. 2013; Perez-Reyes et al. 1988; Ramaekers et al. 2011), while others indicate that effects are less-than-additive (Ballard and de Wit 2011). Clarifying whether effects are synergistic (as compared to additive or less-than-additive) would be valuable for understanding the public health implications of increasing rates of simultaneous alcohol and cannabis use.

It is well-established that both alcohol and cannabis independently affect cognitive performance. Acute and chronic alcohol use is known to confer significant impairments within the domains of memory, decision making, inhibitory control, attention, and psychomotor performance (Bernardin et al. 2014; Caballeria et al. 2020; Evert and Oscar-Berman 1995; Jauhar et al. 2014). In terms of memory function, alcohol most prominently impairs encoding of episodic memories (White 2003) and working memory (Spinola et al. 2022). Cannabis has been most consistently associated with decrements in verbal learning, memory, and attention (Broyd et al. 2016; Gowin et al. 2025). However, existing data are limited and somewhat conflicting, as some studies show minimal impacts of cannabis on cognitive function (Wieghorst et al. 2022). The mechanisms through which cannabis may disrupt memory are not fully understood. The primary psychoactive cannabinoid in the cannabis plant is delta-9-tetrahydrocannabinol (THC) (Pertwee 2014), and evidence suggests that THC may impede memory encoding (Ranganathan et al. 2017) by activating CB1 receptors in the hippocampus and prefrontal cortex, regions which both have dense CB1 receptor expression (Egerton et al. 2006; Wise et al. 2009). While the exact mechanisms underlying the enhanced risks associated with co-use remain unclear, both alcohol and THC can act as central nervous system depressants, with GABA signaling as a potential shared pathway (Cifelli et al. 2020; Lobo and Harris 2008). The limited existing literature on the effects of co-use has indicated that it is associated with impairments in learning, memory, and attention (Bedillion et al. 2021; Deniel et al. 2021; Wade et al. 2020), but the extent to which these impairments differ from single-substance use remains unknown. Variability in study designs (e.g., dosing procedures, timing/order of use of alcohol, and cannabis during the experiment) and differences in participant characteristics limit our ability to draw definitive conclusions, underscoring the need for more research on the mechanism(s) of co-use (Bedillion et al. 2021, 2024).

One important limitation of prior research on alcohol and cannabis co-use—which may contribute to a lack of consilience in findings—is the fact that in most laboratory research on alcohol and cannabis co-administration, participants consumed cannabis containing low levels of THC (e.g., < 7% THC (Hartman et al. 2016), ≤ 3% THC (Downey et al. 2013; Lukas et al. 1992; Perez-Reyes et al. 1988), 11% THC (Ramaekers et al. 2011). Contemporary legal-market cannabis flower products typically contain at least 20% THC (Vergara et al. 2017; Zoorob 2021), and cannabis concentrates contain up to 95% THC (Bidwell et al. 2021). To our knowledge, there are no published co-administration studies that have tested the effects of alcohol combined with legal-market cannabis products containing high levels of THC.

The present study aims to fill existing research gaps through leveraging the well-established mobile laboratory approach developed by researchers in Colorado to study the effects of legal market cannabis products while remaining compliant with state and federal law (Bidwell et al. 2020, 2022; Drennan et al. 2021; Karoly et al. 2022a). Using a within-subjects design, we compared the acute effects of alcohol administered alongside legal-market cannabis (note: cannabis was self-administered by participants *ad libitum*) versus alcohol administered alone on verbal list learning (assessed with the Rey Auditory Verbal Learning Test [RAVLT]) in a laboratory setting. We hypothesized that after using both alcohol and cannabis, individuals would show greater performance decrements compared to when they consumed only alcohol. We also tested whether these relationships differed for males and females.

## Methods

### Participants

Methods were approved by Colorado State University’s Institutional Review Board. Participants were recruited via flyers, word of mouth, and internet advertisements. Eligibility criteria included the following: (1) between ages 21–60; (2) reports heavy drinking over the past three months; defined as more than 4 drinks on any day or more than 14 drinks per week for men; more than 3 drinks on any day or more than 7 drinks per week for women (3) frequent flower cannabis use over the past three months, defined as use at least 3 times per week (note: reporting the use of other forms of cannabis, such as concentrates or edibles, *in addition* to flower did not disqualify individuals from participating as long as they also reported using cannabis flower at least 3 times per week); (4) no daily tobacco use; (5) no history of significant mental health disorders (e.g., bipolar disorder, schizophrenia); (6) no current or history of a substance use disorder and not currently seeking treatment for a substance use disorder; (7) not currently pregnant, breastfeeding or trying to become pregnant; (8) no current use of psychotropic medications besides antidepressants; (9) no illicit drug use in the past 60 days (verified by self-report and urine drug screen); (10) no major medical conditions that contraindicate the use of alcohol or cannabis; (11) no current immune or GI disorder; (12) no use of probiotics or antibiotics in the past 3 months^1^. Participants also had to be willing to abstain from cannabis use for 14 days during the study.

Participants completed three study visits: a baseline session in our on-campus laboratory and two visits in our mobile laboratory (an Alcohol Only session and a Cannabis + Alcohol session), which traveled to the participant’s residence and parked in front of their home for the duration of each session. The mobile laboratory method follows well-established procedures that were developed to allow researchers to study legal-market cannabis while ensuring compliance with state and federal laws, which prohibit bringing legal-market cannabis products onto a university campus (Bidwell et al. 2020, 2022; Drennan et al. 2021; Karoly et al. 2022a, b). Mobile lab methods were adapted by our team to study acute co-administration of alcohol alongside legal-market cannabis (Karoly et al. 2022a, b). All study visits were separated by two weeks. The order in which sessions Alcohol Only and Cannabis + Alcohol were completed was counterbalanced across participants.

### Baseline appointment

All study procedures were administered by a trained research assistant. Participants completed informed consent procedures, received a breathalyzer test, and completed a urinalysis to confirm they did not have illicit substances (besides cannabis) in their system. Females were given a urine pregnancy test. A positive breathalyzer resulted in the appointment being rescheduled. A positive urine test would result in participants being withdrawn from the study. Participants completed a survey that included demographics, medical and mental health history, health behaviors, and substance use. At the end of the visit, participants were given their appointment order, as well as instructions for preparing for the next visit. Participants were asked to abstain from cannabis for the two weeks leading up to the Alcohol Only session. They were not instructed to change their alcohol use during this time (i.e., they could use as much as they wished). In the two weeks leading up to the Cannabis + Alcohol session, participants were instructed to use both cannabis and alcohol as they normally would. The request for participants to refrain from cannabis use during the two weeks prior to the Alcohol Only session was related to one of the larger study aims (Pince et al. 2025), focused on exploring whether participants who typically co-use alcohol and cannabis would change their alcohol use if they reduced or stopped using cannabis. The present analysis does not include data from the two-week periods prior to each laboratory session.

### Mobile lab session—alcohol only

Participants were asked to refrain from alcohol use for 24 h prior to the mobile laboratory visit. They had already been asked to refrain from using cannabis during the preceding two-week period and were reminded again not to use cannabis within 24 h of the visit. At the start of the session, participants completed a breathalyzer, pregnancy and drug test, and the Clinical Institute Withdrawal Assessment Scale (CIWA). Participants then completed a short questionnaire followed by trials 1–5 (principal list), the interference trial (which was note not analyzed in the present study), and the short delay recall trial of the Rey Auditory Verbal Learning Test (RAVLT). This will be referred to as the Alcohol Only, Pre-Use Timepoint. Over the next 20 min, participants completed additional measures relevant to the study’s larger aims and then completed the RAVLT long delay recall trial. Afterwards, participants underwent a blood draw to collect biomarkers to be analyzed for the larger study (results to be reported elsewhere). After completing these procedures, a priming drink was prepared based on the Widmark formula, a calculation that uses weight and sex assigned at birth to determine the alcohol volume necessary to raise blood alcohol content by a certain amount (0.03 g/dL) (Brouwer 2004). Approximately 10 min after receiving the priming drink (referred to as the Alcohol Only, Post-Use Timepoint), participants were given a new principal word list and completed trials 1–5. They were given a new interference list and completed an interference trial and a short delay recall trial. They were asked to recall the principal list 20 min later. Participants then participated in an alcohol self-administration session relevant to the larger study aims and reported elsewhere (Pince et al. 2025).

### Mobile lab session—cannabis + alcohol

Participants were reminded not to use cannabis or alcohol for at least 24 h prior to the visit. All procedures in the Cannabis + Alcohol session are identical to those described for the Alcohol Only session until after the blood draw. At that point in the visit, participants in the Cannabis + Alcohol session were asked to enter their home and use a legal-market flower cannabis product (which they had acquired at a local dispensary without the researchers’ involvement) *ad libitum* while researchers remained outside their residence in the mobile lab. Participants were given a scale and asked to weigh the cannabis product before and after using it. Participants also reported the product/strain name and THC content of each product used. Following cannabis self-administration, participants returned to the mobile lab for a second blood draw. The rest of the procedures were identical to the Alcohol Only session. The first RAVLT administration occurred prior to receiving the priming drink and self-administering cannabis (at the Cannabis + Alcohol Pre-Use Timepoint, which occurred at the same point in the session as the Alcohol Only, Pre-Use Timepoint in the Alcohol Only session). The Post-Use RAVLT administration occurred approximately 20–25 min post-cannabis use and approximately 10 min after receiving the priming drink (Cannabis + Alcohol Post-Use Timepoint). The timing of the Post-Use RAVLT administration was designed to ensure that participants would be feeling the subjective effects of cannabis during the immediate recall trials and the short and long delay recall trials, as prior research suggests that people who use cannabis recreationally experience peak subjective effects (e.g., feeling high) within a few minutes of inhalation and subjective effects remain elevated for at least an hour after smoking (Hunault et al. 2014).

### Measurement of plasma THC concentration

After each session, plasma was extracted from blood samples. Note that one sample was collected during the Alcohol Only session (near the beginning of the session) and two samples were collected during the Cannabis + Alcohol session (one was collected near the beginning of the session, and one was collected following cannabis self-administration). Plasma was stored at −80 °C until the completion of the study, when all plasma was analyzed for THC content in a single batch. High Performance Liquid Chromatography-Tandem Mass Spectrometry was used to assay plasma THC concentration (Conner et al., under review; Dooley et al., under review; Henthorn et al. 2024). A detailed description of the processing methods used in cannabinoid assays is included in the Supplementary Materials.

### Measures

#### Baseline demographics and psychological functioning

At baseline, participants completed a demographics questionnaire, which collected information about age, sex, gender, race, ethnicity, education, and income. Participants also completed the 10-item **The Alcohol Use Disorders Identification Test (AUDIT**) (α = 0.8), which is a well-validated screening tool used to identify hazardous or risky drinking (Saunders et al. 1993), and the 10-item the **Marijuana Dependence Scale (MDS)** (α = 0.8), which measures severity of cannabis dependence (Stephens et al. 2000). Recent substance use was also measured at baseline using a 60-day Timeline Followback (Sobell et al. 1979).

#### **Rey auditory verbal learning test (RAVLT) (**Schmidt 1996**)**

Participants completed the RAVLT twice at both laboratory sessions. The RAVLT was selected as the cognitive performance assessment for this study because it is well-validated and reliable (de Sousa Magalhães et al. 2012), is widely used in alcohol and cannabis use research (Herrera et al. 2024; Ioime et al. 2018; Smith et al. 2017; Takagi et al. 2011; Wagner et al. 2010), and was found by a recent systematic review to be among the top four neuropsychological tests that were most sensitive to cannabis consumption (Stefanidis et al. 2024). The RAVLT involves participants learning words from a “principal list” containing 15 words, which are pre-recorded and played out loud by a computer. After the list is read to them, participants are immediately asked to recall as many words from the list as they can. This procedure is repeated 5 times using this same list (immediate recall trials 1–5). Next, a new list (the interference list) containing 15 different words is read to participants, and they are immediately asked to recall as many as they can. Immediately after this, they are asked to recall the original list—this is the “short delay” recall trial (trial 6). Twenty minutes later, they are again asked to recall the original list, which is called the “long delay” recall trial (trial 7).

At each session, this entire procedure was repeated twice, using different sets of principal and interference lists. At the Alcohol Only session, participants received the principal list immediately after finishing the surveys (which we will refer to as Alcohol Only, Pre-Use Timepoint) and did the long delay recall 20 min after the short delay recall trial. They received the next principal list approximately 10 min after taking their priming drink (Alcohol Only, Post-Use Timepoint) and did the next long delay recall 20 min after the short delay recall trial. At the Cannabis + Alcohol session, participants received their first principal list immediately after finishing the surveys (Cannabis + Alcohol, Pre-Use Timepoint) and did the long delay recall 20 min after the short delay recall trial. They received the next principal list approximately 20–25 min after cannabis self-administration procedures were complete, and as in the Alcohol Only session, this occurred approximately 10 min after the priming drink (Cannabis + Alcohol, Post-Use Timepoint). Participants again did the long delay recall 20 min after the short delay recall trial. Thus, the only difference between sessions is that between the Pre and Post-Use Timepoints at the Alcohol Only session, participants only consumed alcohol, but between the Pre and Post-Use Timepoints at the Cannabis + Alcohol session, they consumed both alcohol and cannabis. We administered 4 distinct sets of principal and interference lists from the RAVLT manual (lists AB, Cr-AB, Ge-AB, and CD). To control for any potential differences between lists, the order in which participants received the lists was counterbalanced across participants. All participants received all 4 lists, but in different orders.

To contextualize RAVLT performance at each study condition, we used an established criterion of 1.5 *SD* below the normative mean (consistent with prior work (Beier et al. 2019) as an indicator of learning and memory impairment. We used normative means from the Mayo Clinic Study of Aging (Roberts et al. 2008; Stricker et al. 2021). Because the Mayo study only includes individuals over age 30, the 30–39 year old norms for those under age 30 in this sample.

### Data analysis plan

Analyses were conducted with R software (version 4.3.1). We used the lme4 package to conduct three separate linear mixed-effects models for immediate recall, short delay recall, and long delay recall. For the immediate recall model, we included a 3-way interaction of condition (Alcohol Only Pre-Use, Alcohol Only Post-Use, Cannabis + Alcohol Pre-Use, Cannabis + Alcohol Post-Use), RAVLT trial (1, 2, 3, 4, 5), and sex (male, female). We chose to include the effects of RAVLT trial (rather than taking a sum of the first 5 trials, which is also a common approach for analyzing immediate recall on the RAVLT (Bassir Nia et al. 2022) as it provides more information about how the pattern of initial learning may differ across conditions. Our chosen approach is consistent with prior RAVLT research (Estévez-González et al. 2003). The immediate recall model included covariates for education (high school or less, some college, or bachelor’s degree or higher), household income (less than $30k, greater than or equal to $30k), age, and tolerance (i.e., yes/no response to MDS item #1, “The need to smoke more marijuana to achieve the same “high”). Subject was specified as a random intercept. Covariates were selected to account for sociodemographic and individual differences that could influence RAVLT performance. For the short delay recall model and the long delay recall analyses, we tested a 2-way interaction of condition and sex, and included the same covariates as the immediate recall model. For all three models, Alcohol Only Pre-Use was specified as the reference category, and pairwise comparisons between conditions were examined with Tukey’s post hoc test. To assess the significance of model terms, we used the Anova function to examine Wald F tests, applying Type II with the Kenward-Roger degrees of freedom approximation. To facilitate comparisons between RAVLT performance in each condition and normative RAVLT performance, we calculated the percentage of participants in the current study who scored at least 1.5 *SD* below the normative mean for total immediate recall (the sum of the first 5 trials), short delay recall, and long delay recall at each study condition.

We used Spearman’s rank correlation to examine potential effects of THC in blood plasma at the Alcohol Only session, Pre-Use Timepoint, when participants did not consume cannabis during the session, and total words recalled during the first 5 timepoints to determine whether cannabis usage outside the study session may have affected performance. We also used Spearman’s rank correlation to examine the association between change in THC between the Pre and Post-Use Timepoints at the Cannabis + Alcohol session (after cannabis use) and change in words recalled between the Pre and Post-Use Timepoints at the Cannabis + Alcohol session to determine if individuals with greater change in THC after using cannabis showed a corresponding greater decrease in RAVLT performance.

Lastly, as a sensitivity analysis, we repeated all three models, including days of cannabis use and days of alcohol use reported on the TLFB at baseline as covariates to explore potential effects of alcohol and cannabis use history on RAVLT performance.

## Results

### Participant characteristics

The sample (*N* = 60) was mostly White (89.8%), non-Hispanic (81.4%), with a mean age of 30.4 years (*SD* = 9.3). The males (*n* = 36) and females (*n* = 24) did not differ in racial or ethnic identity, or in age, education, or income (all *p* >.05). On the Timeline Followback administered at baseline, males and females both used cannabis on most days (*median* > 75%) and used both alcohol and cannabis on about a quarter of days. See Table 1 for additional participant demographics.

**Table 1:** Participant demographics

	Male (*N* = 36)	Female (*N* = 24)	*P*-value
Race			
White	30 (83.3%)	23 (95.8%)	0.65
Black	3 (8.3%)	0 (0%)	
Asian	1 (2.8%)	0 (0%)	
Other/Mixed	2 (5.6%)	1 (4.2%)	
Ethnicity			
Hispanic	5 (13.9%)	6 (25.0%)	0.32
Age			
Mean (SD)	31.7 (9.79)	28.4 (7.95)	0.14
Income			
<$30k	15 (41.7%)	13 (54.2%)	0.43
* ≥*$30k	21 (58.3%)	11 (45.8%)	
Education			
High School or Less	4 (11.1%)	1 (4.2%)	0.34
Some College or Associates Degree	19 (52.8%)	10 (41.7%)	
Bachelor’s Degree or Higher	13 (36.1%)	13 (54.2%)	
AUDIT			
Mean (SD)	10.8 (4.87)	8.92 (5.27)	0.07
MDS Score			
Mean (SD)	2.6 (2.4)	3.1 (2.3)	0.30
MDS Q#1 “The need to smoke more to achieve the same high”			
No	23 (63.9%)	11 (45.8%)	0.19
Yes	13 (36.1%)	13 (54.2%)	
TLFB Days Using Cannabis (%)			
Median [Min, Max]	78.3 [5.0, 100.0]	75.8 [11.6, 98.3]	0.61
TLFB Days Using Alcohol and Cannabis (%)			
Median [Min, Max]	25.8 [0, 98.3]	24.2 [0, 98.3]	0.69
TLFB Days Using Alcohol (%)			
Median [Min, Max]	35.8 [0, 100.0]	30.8 [0, 98.3]	0.33
TLFB Drinks per Drinking Day			
Median [Min, Max]	3.9 [1.9, 8.1]	2.9 [0.2, 8.2]	0.10
Missing	4 (11.1%)	1 (4.2%)	
Alcohol Only Session BrAC (g%)			
Mean (SD)	0.023 (0.01)	0.017 (0.01)	0.03*
Cannabis + Alcohol Session B BrAC (g%)			
Mean (SD)	0.019 (0.01)	0.018 (0.01)	0.74
Missing	1 (2.8%)	0 (0%)	
THC level (ng/mL)			
Alcohol Only, Pre-Use Median [Min, Max]	2.84 [0, 74.8]	1.03 [0, 11.0]	0.10
Missing	6 (16.7%)	4 (16.7%)	
Cannabis + Alcohol, Pre-Use Median [Min, Max]	2.84 [0, 98.2]	0.820 [0, 45.2]	0.28
Missing	3 (8.3%)	5 (20.8%)	
Cannabis + Alcohol, Post-Use Median [Min, Max]	81.2 [1.56, 686]	49.9 [2.64, 835]	0.28
Missing	2 (5.6%)	6 (25.0%)	

*AUDIT* Alcohol Use Disorders Identification Test, *BrAC* Breath Alcohol Concentration, *MDS* Marijuana Dependence Scale, *TLFB* Timeline FollowBack

*significant (*p* <.05 difference between males and females

The average THC% of flower cannabis used during the study was 22.6%. Participants used cannabis on an average 8.1 days in the two weeks prior to the Cannabis + Alcohol session, and on an average of 2.5 days in the two weeks prior to the Alcohol Only session (indicating that participants were not abstinent during the two-week period when they were asked to refrain from cannabis use).

At the pre-substance use timepoints, most participants did not show learning/memory impairments on the RAVLT. Specifically, at the Alcohol Only Pre-Use Timepoint, 19.4% of participants scored at least 1.5 *SD* below the normative mean for total immediate recall, 9.7% scored at least 1.5 *SD* below the normative mean for short delay recall, and 16.10% scored at least 1.5 *SD* below the normative mean for long delay recall.

### Breath alcohol concentration and plasma THC concentration

Breath alcohol after the priming drink was slightly below target levels of 0.03 g% for the Alcohol Only session (*mean* = 0.02 g%, *SD* = 0.01), and females had lower breath alcohol levels relative to males (*p* =.03). Levels were also below target levels for the Cannabis + Alcohol session (*mean* = 0.02 g%, *SD* = 0.01), but there was no difference between males and females. Plasma THC concentration was similar for the Alcohol Only Pre-Use (*median* = 1.9 ng/mL) and Cannabis + Alcohol Pre-Use Timepoints (*median* = 2.5 ng/mL) but were significantly higher for the Cannabis + Alcohol Post-Use Timepoint (*median* = 61.6 ng/mL), after participants used cannabis in their homes. Males and females did not differ in terms of plasma THC concentration at any of the timepoints (Table 1).

### Rey verbal learning test results

#### Immediate recall

**Fig. 1:**
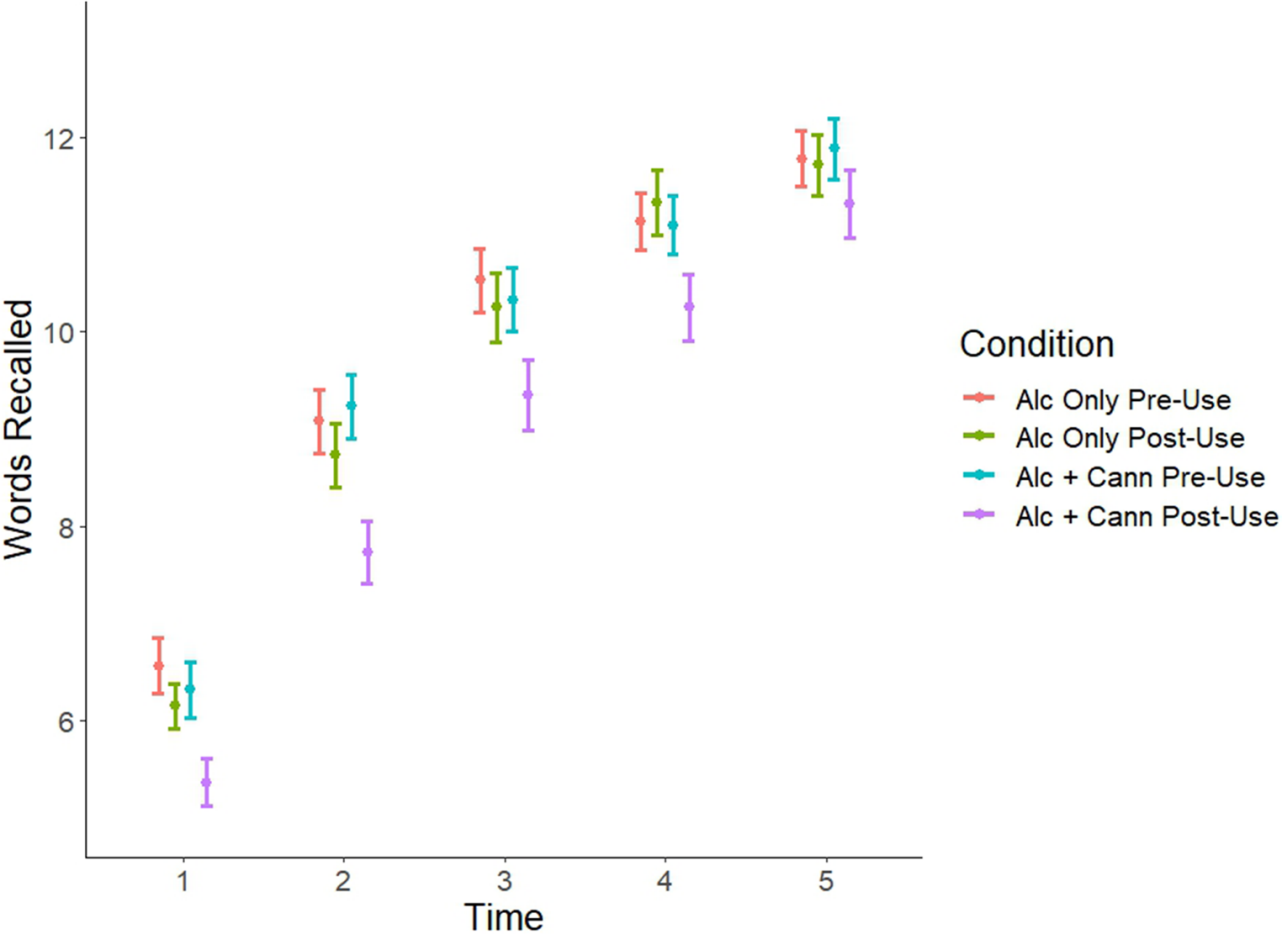
Words Recalled in Immediate Recall Trials. Figure 1 depicts the number of words correct on the RAVLT at trials 1–5. The red, green, blue, and purple lines represent each study condition. Error bars represent the standard error of the mean

**Fig. 2:**
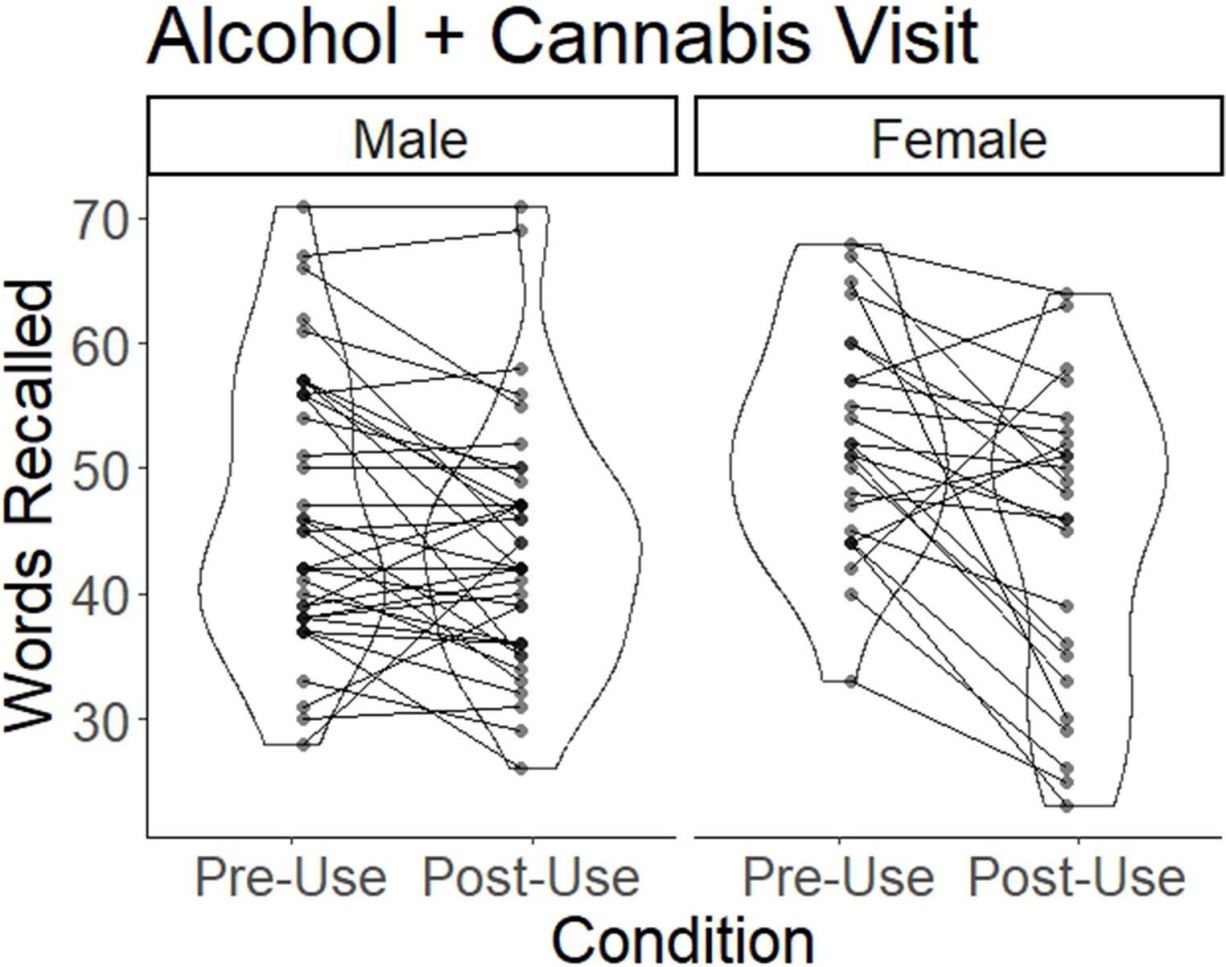
Total Words Recalled on RAVLT Trials 1–5 By Sex. Individual lines represent each participant’s number of words recalled (i.e., sum of words recalled in trials 1–5) at the Pre and Post-Use Timepoints at the Cannabis + Alcohol session. The violin plots represent the distributions of the data for each condition. Data are presented separately for males and females

Participants recalled an average of 63.4% of words across the first five trials (*SD* = 21%). This amounts to 47.5 words out of a possible 75 over the 5 trials (15 words per trial). There was a main effect of RAVLT trial (*F*_4,1102_ = 405.5, *p* <.001), where participants recalled more words as the task went on, starting at 40.7% (6.1 out of 15) at trial 1 and finishing at 78.0% (11.7 out of 15) at trial 5. There was a main effect of condition (see Fig. 1; Table 2, *F*_3,1102_ = 23.7, *p* <.001), where participants recalled fewer words in the Cannabis + Alcohol Post-Use condition (*mean* = 58.6% [44 words], *SD* = 21.6%) compared to Alcohol Only Post-Use *(mean* = 64.9%, [48.7 words] *SD* = 20.5%). There was a sex-by-condition interaction (*F*_3,1102_ = 4.6, *p* =.003), where females showed a greater decrease in the Cannabis + Alcohol condition relative to males (Fig. 2). There was no condition by time interaction. There was a main effect of income (*F*_1,53_ = 6.8, *p* =.01; higher income was associated with more words remembered) (see Table 2). Tukey’s post hoc tests showed that participants recalled approximately one fewer word per trial in the Cannabis + Alcohol Post-Use condition relative to all 3 other conditions (*p* <.001), but there were no other condition differences.

**Table 2:** Model results during the immediate recall phase

	F	df	df.res	*p*
RAVLT trial	405.53	4	1102	< 0.001 ***
Condition	23.68	3	1102	< 0.001 ***
Sex	2.56	1	53	0.12
Age	1.28	1	53	0.26
Education	1.28	2	53	0.28
Income	6.83	1	53	0.01 *
Tolerance	0.28	1	53	0.60
RAVLT Trial x Condition	0.82	12	1102	0.63
RAVLT Trial x Sex	0.81	4	1102	0.52
Condition x Sex	4.60	3	1102	0.003 **
RAVLT Trial x Condition x Sex	0.59	12	1102	0.85

Analysis of Deviance Table (Type II Wald F tests with Kenward-Roger degrees of freedom), using *Words Recalled* as the response variable

Significance codes: ***p <.001, **p <.01, *p <.05

At the Alcohol Only Post-Use Timepoint, 22.6% of participants scored at least 1.5 *SD* below the normative mean for total immediate recall. At the Cannabis + Alcohol Post-Use Timepoint, 35.5% of participants scored at least 1.5 *SD* below the normative mean for total immediate recall.

### Short delay recall trial

**Fig. 3:**
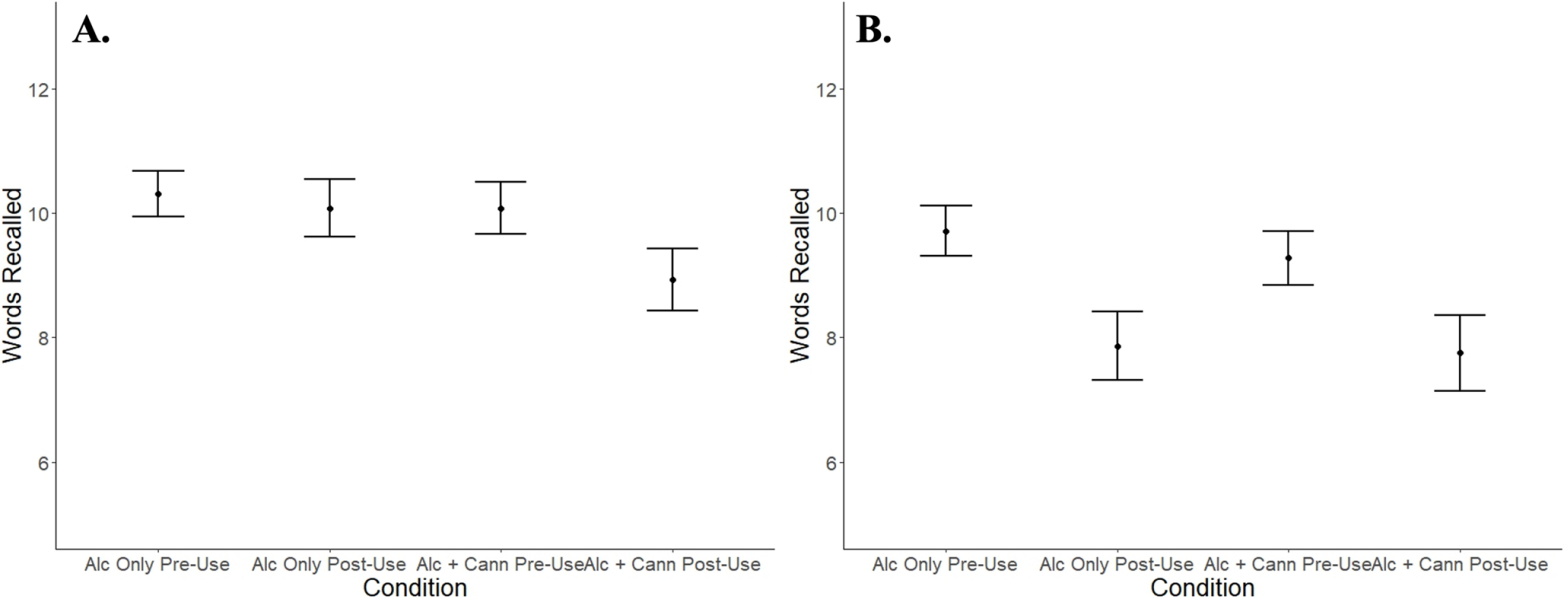
Words Recalls in Delay Recall Trials. Figure 3 depicts the number of words correct on the RAVLT short delay (panel A) and long delay (panel B) recall trials for each study condition. Error bars represent the standard error of the mean

Overall, participants recalled an average of 65.6% of words (9.84 out of 15 words) on the short delay recall trials. There was a main effect of condition (*F*_3,174_ = 4.5, *p* <.001), but no condition-by-sex interaction (see Fig. 3; Table 3). Post-hoc analysis showed that the Cannabis + Alcohol Post-Use condition was associated with recall of 1.15 to 1.38 fewer words relative to the other three conditions (*p* <.05), but none of the other three conditions differed. At the Alcohol Only Post-Use Timepoint, 19.4% of participants scored at least 1.5 *SD* below the normative mean for short delay recall. At the Cannabis + Alcohol Post-Use Timepoint, 24.4% of participants scored at least 1.5 *SD* below the normative mean for short delay recall.

**Table 3:** Model results during the short and long delay recall phases

	Short Delay		Long Delay	
	F	*p*	F	*p*
Condition	4.5	0.005 **	8.6	< 0.001 ***
Sex	2.5	0.12	3.0	0.09
Age	3.2	0.08	1.9	0.17
Education	0.9	0.38	0.9	0.40
Income	2.3	0.14	2.8	0.10
Tolerance	0.1	0.82	0.0	0.84
Condition x Sex	1.1	0.35	0.4	0.74

Analysis of Deviance Table (Type II Wald F tests with Kenward-Roger degrees of freedom), using *Words Recalled* as the response variable

Significance codes: ***p <.001, **p <.01, *p <.05

### Long delay recall trial

Participants recalled an average of 57.6% of words (8.64 out of 15 words) after the long delay. There was a main effect of condition (*F*_3,174_ = 8.66, *p* <.001, see Fig. 3; Table 3). Post hoc analysis showed that participants recalled fewer words in both Alcohol Only Post-Use and Cannabis + Alcohol Post-Use conditions relative to Alcohol Only and Cannabis + Alcohol Pre-Use conditions (1.5 to 2 fewer words, *p* <.01), but the Pre-Use conditions did not differ from each other, and the Alcohol Only and Cannabis + Alcohol Post-Use conditions did not differ from each other. There were no interactions with sex. At the Alcohol Only Post-Use Timepoint, 40.3% of participants scored at least 1.5 *SD* below the normative mean for long delay recall. At the Cannabis + Alcohol Post-Use Timepoint, 37.1% of participants scored at least 1.5 *SD* below the normative mean for long delay recall.

### Association between THC levels and words recalled

At the Alcohol Only session, following a period where participants were instructed to abstain from cannabis for two weeks, there was a trend for an association between higher plasma THC concentration and words recalled (rho = 0.24, *p* =.09, *N* = 50 due to missing blood samples). At the Cannabis + Alcohol session, there was a trend for an association between greater number of words recalled after cannabis use and greater increase in plasma THC concentration after cannabis use (rho = 0.26, *p* =.07, *N* = 49 due to missing blood samples).

### Sensitivity analysis

For immediate recall, neither days of alcohol use nor days of cannabis use were associated with the number of words recalled, and the inclusion of these variables did not change the significance of condition or the sex-by-condition interaction. For short delay recall and long delay recall, neither days of alcohol nor cannabis use were associated with the number of words recalled, and the inclusion of these variables did not alter the significant findings.

## Discussion

The present study indicates that the common practice of combining alcohol with legal-market cannabis products containing high amounts of THC (Stevens et al. 2021) may confer risk learning and memory. Further, the negative effects of cannabis on initial memory encoding appear to be exaggerated for females as compared to males. Specifically, we found that co-administration of alcohol and legal-market cannabis (at naturalistic, participant-selected doses) leads to greater impairment in immediate recall of verbal information than alcohol administered without cannabis. The Alcohol Only and the Cannabis + Alcohol conditions showed similarly reduced recall during the long delay trial at the Post-Use Timepoints compared to Pre-Use. Females showed greater impairments in the Cannabis + Alcohol condition than males for immediate recall trials, but no sex differences emerged for delayed recall trials. Comparisons to RAVLT normative data indicate that before alcohol and cannabis use, 10–20% of participants across each trial type showed impaired learning and memory (defined as being more than 1.5 *SD* below the normative mean), but in the Post-Use conditions, these percentages increased to 20–40%, with the greatest number of participants showing impairment in recall after the long delay.

These findings are consistent with prior research demonstrating that cannabis impairs verbal memory (Broyd et al. 2016) and that co-administration of alcohol alongside cannabis is associated with greater cognitive impairment compared to alcohol administered on its own (Chait and Perry 1994; Deniel et al. 2021; Wickens et al. 2022). Findings are also in line with prior research using the mobile laboratory method, which has shown that naturalistic, participant-selected doses of cannabis confer significant cognitive impairment (particularly in the domain of verbal memory) (Bidwell et al. 2020). Our finding that immediate and delayed recall performance was reduced following cannabis use compared to during a sober RAVLT administration (the Pre-Use Timepoints) is also consistent with prior work (Ranganathan et al. 2017). Interestingly, we found trend-level associations between plasma THC concentration and the number of words recalled. This finding could reflect self-titration (i.e., modulating inhalation length/depth to achieve desired effects (Leung et al. 2021). However, the lack of association between tolerance or days of cannabis use and words recalled suggests that individual differences in tolerance or experience may not fully explain this finding. Although not assessed here, there is also evidence that age of onset of cannabis use may impact neurocognitive function (Becker et al. 2010), and could also impact heaviness of use, so future studies may address this possibility.

Notably, we observed differences in immediate recall between the Alcohol Only Post-Use and Cannabis + Alcohol Post-Use conditions, but these differences were no longer present after the long delay. While cannabis and alcohol co-use may confer particular risk for initial learning, it is also possible that features of our design may have contributed to this finding. Specifically, in the Post-Use conditions, the RAVLT was administered approximately 10 min after the priming drink. This timing was selected to facilitate the testing of other study aims. Given that peak blood alcohol levels occur approximately 30–60 min after oral alcohol ingestion (Plawecki et al. 2019), participants likely had not yet reached peak alcohol effects by the time the immediate recall trials took place. However, participants were likely experiencing increased subjective effects of alcohol by the time of the long delay recall trial. It is plausible that the similarity in performance across the two Post-Use conditions during the long delay trial reflects the heightened effects of alcohol over time. By the time of the long delay recall trial, the alcohol effects may have reached a level that masked or overshadowed any additional impairment attributable to cannabis. Future research could adjust the timing of alcohol and cannabis relative to RAVLT administration to explore this possibility. Fatigue at the time of the delayed recall task may have also contributed to these results; this possibility could be tested with the inclusion of a sober control condition.

In the present study, females had greater impairments in verbal memory following alcohol and cannabis co-use than males, but only for immediate recall trials, perhaps reflecting sex-specific vulnerabilities in early encoding or attentional processes. Another explanation may be that the increased cognitive load for the delayed recall trials, combined with potential participant fatigue after completing multiple trials of the task, may dilute sex-related differences in performance. Alternatively, males and females may tend to use different cognitive strategies (Wang and Carr 2014) during encoding and retrieval, and cannabis may selectively impair strategies more often employed by females during immediate recall. The present findings are partially consistent with a recent study in which intravenous THC (at either a high dose, low dose, or placebo) was administered to human participants prior to completing the RAVLT (Bassir Nia et al. 2022), and no sex effects or sex by dose interactions were observed for immediate or delayed recall. The discrepancy in immediate recall findings may be due to the different route of THC administration or differences between controlled and naturalistic dosing procedures. More broadly, the existing data on cannabis related sex differences on cognition are mixed, with some studies suggesting that males are more vulnerable to cognitive impairment following cannabis use (Hirst et al. 2021; Martin et al. 2021), some data suggesting that females are more vulnerable (Pope et al. 1997) and other studies indicating little evidence for the presence of sex differences (Arkell et al. 2022).

Currently, we lack a precise understanding of the specific mechanisms through which the combined pharmacological effects of alcohol and cannabis may heighten cognitive impairment. One mechanism may involve the GABA system—a shared target of both alcohol and THC (Friend et al. 2017; Lobo and Harris 2008), which is known to underlie memory functions (Gasbarri and Pompili 2014). It is possible that combining alcohol and cannabis amplifies activation of GABA_A_ (which is specifically implicated in memory encoding and consolidation processes (Chapouthier and Venault 2002; Kim et al. 2012) to produce decrements in learning and memory functions. Another potential mechanism relates to the overlapping neuropharmacological effects of cannabis and alcohol on the prefrontal cortex. Both alcohol and cannabis have been shown individually to confer disruptions within this region (Abernathy et al. 2010; Egerton et al. 2006), and co-use may exacerbate these disruptions to confer additive impairment to prefrontally-mediated cognitive functions, including learning and memory (Miller and Constantinidis 2024; Peters et al. 2013). Co-use may also exert additive or synergistic effects on memory via overlapping effects on the hippocampus. Alcohol impairs synaptic plasticity (Avchalumov and Mandyam 2020), and cannabis impairs memory function via CB1 receptor activity (Wise et al. 2009). Thus, co-use may amplify hippocampal dysfunction, resulting in compounded memory impairment. Although the current results do not address mechanisms, our findings underscore the need for additional research to delineate the neurobiological processes underlying the deleterious effects of combined alcohol and cannabis on learning and memory.

## Limitations and future directions

This study has several limitations of note. First, the sample is mostly white and non-Hispanic, and individuals were not recruited based on alcohol or cannabis use disorder diagnoses. This work should be replicated in more diverse samples and among individuals who report more severe substance use patterns. In addition, the naturalistic mobile laboratory design improves ecological validity compared to previous studies on this topic, but the nature of the method limits experimental control over cannabis doses used during the session. The inclusion of plasma THC measurements (and the fact that plasma THC concentration increased post-cannabis use) serves as a manipulation check (i.e., it verifies that participants did in fact use cannabis during the cannabis use session) to somewhat reduce concerns around the lack of control over THC dose.

In addition, the relatively low dose of alcohol may have precluded our ability to observe alcohol/cannabis interaction effects on memory. Specifically, the dose of alcohol used in the present study may be below what is needed to show significant memory deficits in this population, as other studies of alcohol and cognition have targeted higher BrAC levels (Saults et al. 2007; Schweizer et al. 2006). In addition, cannabis was administered prior to alcohol during the Cannabis + Alcohol session, and it is possible that the order of administration may have impacted results (Gunn et al. 2020). Relatedly, while the timing of the Post-Use RAVLT administration corresponded to significantly elevated subjective effects for cannabis (Hunault et al. 2014), peak alcohol effects likely occurred after the RAVLT administration, due to design constraints related to other study aims. Also, the present study lacked a Cannabis Only session with which to compare the Cannabis + Alcohol findings. Finally, it is possible that individuals could have been experiencing cannabis withdrawal prior to the Alcohol Only session (given that they were asked to refrain from cannabis use in the two weeks leading up to the session).

Future studies could counterbalance the order of alcohol and cannabis administration, use a higher dose of alcohol, adjust timing of the alcohol administration relative to cognitive assessments of interest, assess for withdrawal if participants are asked to reduce or refrain from use of any substances prior to study sessions, and include a Cannabis Only session. These design elements would aid in informing conclusions about whether alcohol and cannabis confer synergistic effects on memory impairment. Finally, we only explored the effects of cannabis and alcohol on verbal memory in the present study. Future work should test the cognitive effects of these substances across other domains. Future studies could also examine additional moderators (e.g., route of administration, THC: CBD ratios) (Gunn et al. 2022), which may influence the effects of cannabis and alcohol use on cognitive outcomes.

## Conclusions

Co-use of alcohol and legal-market cannabis significantly impairs immediate recall of verbal information compared to alcohol administered without cannabis. Our findings suggest that females may be more susceptible to cannabis-related memory deficits than males, but this difference disappears under more demanding task conditions. Results underscore the need for more research on this topic, as gaining a better understanding of the risks associated with the combination of alcohol and legal-market cannabis could inform public health and safety messaging and could have harm-reduction implications for the growing number of individuals who combine alcohol with cannabis.

## Supplementary Information

Below is the link to the electronic supplementary material.

Supplementary Material 1

## Data Availability

Data will be shared on reasonable request to the corresponding author. Data will also be available via the NIAAA Data Archive (NIAAA_DA_) https://nda.nih.gov/niaaa.

## Footnotes

1 Note: criteria 11 and 12 pertain to the gut microbiome and immune biomarker aim of the study, which will be reported in subsequent publications from the larger study.
