# Supplementary material for: Effects of legal-market cannabis and alcohol on verbal learning and memory
Source: Psychopharmacology (Berl). 2025 Sep 25;243(4):805–18. doi: 10.1007/s00213-025-06882-z (PMC13007496; doi:10.1007/s00213-025-06882-z)
Supplement: Supplementary file 1 — Supplementary Material 1 [file 213_2025_6882_MOESM1_ESM.docx]

**Cannabinoid Analysis by LC−MS/MS**

∆^9^-THC and ∆^9^-THC-d_3_ standards were purchased from Cerilliant (Round Rock, TX, USA). Water, methanol, and acetonitrile (LC–MS grade) were purchased from Millipore (Burlington, MA, USA). Formic acid (LC−MS grade) was obtained from Sigma-Aldrich (St. Louis, MO, USA). Captiva EMR-Lipid columns (1 mL, 40 mg) were purchased from Agilent Technologies (Santa Clara, CA, USA). Liquid chromatography columns were purchased from Restek Inc. (Bellefonte, PA, USA).

Matrix-matched calibrators and controls were prepared by the addition of appropriate volumes of methanolic stock standard mixtures (0.01, 0.1,1.0, or 10 µg/mL) to 100 µL of cannabinoid-free plasma to produce calibrators at 0.5, 1, 5, 10, 50, 100, 500, and 1000ng/mL. Quality control samples were prepared at 5 ng/mL, 750, and 700 ng/ml. Quality control samples were run after every 20 subject samples, with an expected accuracy of +/- 20%.

Subject plasma samples, matrix-matched standards, and quality control samples were prepared for LC−MS/MS analysis by protein precipitation and lipid removal. Ten microliters of the internal standard solution (0.3 µg/mL for ∆^9^-THC-d_3_) was added to 100 μL of plasma sample and vortexed in a polypropylene microcentrifuge tube. 600 μL of ice-cold acetonitrile/methanol (85%/15%) was added dropwise while vortexing to precipitate proteins. Samples were centrifuged at 14,000 rpms and supernatants were transferred to Captiva EMR-Lipid columns for lipid removal. Using a positive-pressure manifold, 3 psi of pressure was applied to elute the samples through the columns. Eluents were collected into clean glass test tubes and dried under nitrogen at 45°C. Eluents were reconstituted in 100 µL of water/methanol (50%/50%) with 0.1% formic acid and transferred to autosampler vials with pulled-point inserts for LC−MS/MS analysis.

Samples were analyzed with an Agilent 1290 Infinity II liquid chromatograph coupled to an Agilent 6475 triple quadruple mass spectrometer equipped with an Agilent Jet Stream electrospray ionization source (Agilent, Santa Clara, CA). Chromatography was performed on a Restek Raptor biphenyl column (3.0 × 50 mm, 2.7 μm) and held at 40°C. A sample volume of 10 μL was injected, and a mixture of water with 0.1% formic acid (A) and methanol with 0.1% formic acid (B) was introduced at a flow rate of 0.4 mL/min. Gradient elution started at 40% B, which was increased to 70% B over 1 min and subsequently to 75% B over 3.5 min, and ended at 100% B at 6 min. The ionization source conditions used were as follows: positive polarity, nebulizer pressure of 45 psi; gas flow of 12 L/min at 300°C; sheath gas flow of 12 L/min at 375°C; capillary voltage of 3500 V; and nozzle voltage of 2000V. The ion transitions monitored are displayed in Table 1. ∆^9^-THC was confirmed by the retention time and the product ion ratio (± 20%) correlation between the sample peaks and corresponding standards. Data collection and processing were performed by using Agilent MassHunter quantitative software (v.B.12.01). Quantitation was performed with linear regression using 8-point calibration curves from 0.5 ng/mL to 1000 ng/mL and the limit of detection was 0.25 ng/ml for each analyte.

Any values that were not detected (ND) were replaced with zeros. The limit of quantitation was 0.5 ng/ml for each analyte. Anything below the limit of quantitation was converted to 0.25 ng/ml (LOQ/2).

Table 1. LC−MS/MS ion transitions monitored for ∆^9^-THC in human plasma.

| **Analyte Name** | **Precursor Ion** | **Product Ion** | **Fragmentor (V)** | **Collision Energy (V)** | **Polarity** |
| --- | --- | --- | --- | --- | --- |
| ∆^9^-THC | 315.2 | 193.1 | 122 | 20 | Positive |
| ∆^9^-THC | 315.2 | 123 | 122 | 32 | Positive |
| ∆^9^-THC-d_3_ | 318.2 | 196.1 | 122 | 20 | Positive |
| ∆^9^-THC-d_3_ | 318.2 | 123 | 122 | 32 | Positive |
